# Premedication with dexmedetomidine for prevention of hyperdynamic response after electroconvulsive therapy: a cross-over, randomized controlled trial

**DOI:** 10.1186/s12888-021-03406-9

**Published:** 2021-08-17

**Authors:** Pattika Subsoontorn, Varinee Lekprasert, Punjaporn Waleeprakhon, Pichai Ittasakul, Atchaporn Laopuangsak, Suwimon Limpoon

**Affiliations:** 1 Department of Anesthesiology, Faculty of Medicine Ramathibodi Hospital, Mahidol University, Bangkok, Thailand; 2 Department of Psychiatry, Faculty of Medicine Ramathibodi Hospital, Mahidol University, 270 Rama VI Road, Rachathewi, Bangkok, 10400 Thailand

**Keywords:** Dexmedetomidine, Electroconvulsive therapy, Acute hyperdynamic response

## Abstract

**Background:**

Electroconvulsive therapy (ECT) is an effective therapy for psychiatric disorders, but is associated with acute hyperdynamic responses including transient hypertension and tachycardia. This study aimed to assess the effectiveness of premedication with dexmedetomidine for hemodynamic attenuation after ECT and to evaluate its effects on seizure duration, postictal asystole duration, post ECT agitation and recovery time.

**Methods:**

Twenty-four psychiatric patients who underwent a total of 72 ECT sessions (three sessions per patient) were randomly allocated to receive either dexmedetomidine 0.5 mcg/kg intravenous, dexmedetomidine 1 mcg/kg intravenous, or saline (control group) 15 min before the first ECT session. The patients subsequently received the other two premedication options for their next two ECT sessions. Blood pressure and heart rate were recorded at 5, 10, and 15 min after drug infusion and at 2.5, 5, 7.5, 10, 15, 20, 25, and 30 min after ECT. Asystole duration, seizure duration, post ECT agitation and recovery times were also recorded.

**Results:**

The baseline characteristics were similar between the groups. Systolic blood pressure in both dexmedetomidine groups was significantly lower than that in the control group after ECT (*p* = 0.002). Diastolic blood pressure and heart rate were significantly lower in the dexmedetomidine 1 mcg/kg group (*p* = 0.002 and *p* = 0.013, respectively) compared with the control group. Asystole duration, seizure durations, post ECT agitation and recovery times were similar between the groups.

**Conclusions:**

Dexmedetomidine 1 mcg/kg administered 15 min before ECT attenuated the hemodynamic response, including suppressing the systolic, diastolic and heart rate increases, during ECT without affecting recovery time. It also did not prolong the post-stimulus asystole duration.

**Trial registration:**

TCTR20170715003, registered at Thai Clinical Trials Registry (TCTR), principal investigator: Pattika Subsoontorn, date of registration: 15/07/2017.

## Background

Electroconvulsive therapy (ECT) is an effective therapy for psychiatric disorders in patients who have not responded to pharmacotherapy. In ECT, a generalized seizure is induced by a brief unilateral or bilateral electrical stimulus. ECT causes generalized autonomic nervous system stimulation, initially producing bradycardia induced by parasympathetic nerve stimulation, followed immediately by more prominent sympathetic stimulation that results in transient tachycardia and hypertension [1]. The acute hyperdynamic response may be harmful to patients with ischemic heart diseases, hypertension and cerebrovascular disease [2, 3].

Many drugs, such as α-2 adrenergic agonists, β-blockers and opioids, have been used to attenuate the acute hemodynamic responses typically induced by the ECT stimulus [4–7]. Alpha-2 adrenergic agonists decrease stress-induced sympathetic responses to improve intraoperative hemodynamic stability [8]. Dexmedetomidine is a highly selective α-2 adrenergic agonist. It inhibits central sympathetic outflow at presynaptic receptors and reduces peripheral norepinephrine release [9]. A previous meta-analysis reported reductions in morbidity and mortality associated with various types of surgery when patients were treated with dexmedetomidine [10]. Some studies showed attenuation effect of dexmedetomidine for premedication before ECT but some did not. This may be due to differences in dexmedetomidine dose and anesthetic regimen used during ECT [4–6, 11, 12]. This study aimed to evaluate the effect of dexmedetomidine (0.5 mcg/kg and 1 mcg/kg) on the hyperdynamic response after ECT and to evaluate the effect on seizure duration, postictal asystole duration, post-ECT agitation and recovery time.

## Methods

This study was a crossover, double-blind randomized controlled trial conducted during May 2017 to April 2018 in Ramathibodi Hospital, Mahidol University, Thailand. The study participants were selected from patients recommended for ECT by a psychiatrist, and who were between 18 to 70 years old with an American Society of Anesthesiologist (ASA) physical status of I–III. The exclusion criteria were patients with liver disease, severe ventricular dysfunction, advanced heart block, or an allergy to the study drugs, or who were currently pregnant or using any β-blockers or narcotics. Informed consent was obtained from all eligible patients, or from relatives of patients who were unable to make an informed decision, before enrollment in the study.

After obtaining approval from Ramathibodi Ethics Committee (ID 02–60-15)**,** the study was registered before participant recruitment with Thai Clinical Trials Registry (https://www.thaiclinicaltrials.org/). The registration number is TCTR20170715003, date of registration: 15/07/2017. The sample size was calculated from a previous study [5] to detect a 25% difference in hemodynamic values. A power analysis was performed with a power of 0.8 and significance level of 0.05, and a sample size of 24 patients was required to reach the primary study endpoint.

The primary outcomes were post-ECT blood pressure and heart rate (HR). The secondary outcomes were incidence of post-ECT agitation, seizure duration (motor and EEG), asystole duration (post-ictal and post-stimulus) and recovery time. The asystole duration was determined by the absence of ventricular contraction detected on an electrocardiogram. Patients enrolled in the study were randomly assigned by computer-generated random numbers to receive one of three premedications that were given 15 min before their first ECT session. The patients subsequently received the other two premedication options for their next two ECT sessions. After randomization, sequence pattern for each patient would be enclosed in envelope. Patients were blinded from the study drugs. The drug solutions were prepared by an anesthesiologist who was involved in the study, but administration was performed by an anesthetic nurse who was blinded to the study drugs.

Each patient was evaluated by an anesthesiology resident before the procedure. Baseline characteristics of the patients, including name, height, weight, comorbidities, current medication and ASA physical status, were recorded. If the patient was on antihypertensive medication, it was continued as usual before the ECT. After standard monitoring, patients in group A received dexmedetomidine 0.5 mcg/kg, patients in group B received dexmedetomidine 1 mcg/kg and patients in the control group received saline. All solutions were prepared in a total volume of 25 mL and given by intravenous (IV) infusion over 15 min. Arterial blood pressure and HR were recorded immediately before, and at 5, 10 and 15 min after starting the drug infusion.

Immediately after the drug infusion was completed, anesthesia was induced with thiopental 2–3 mg/kg or propofol 1–2 mg/kg. The total dose of propofol or thiopental that each patient received was recorded. After loss of consciousness, a pneumatic tourniquet was applied to one leg and inflated to isolate the leg circulation and allow for an accurate assessment of the motor seizure. Succinylcholine (1–2 mg/kg, IV) was then administered and ventilation was assisted with 100% oxygen in all patients. When paralysis was achieved, the electrical stimulus was applied. After cessation of the EEG and clinical motor seizure, manual ventilation was initiated until the patient’s spontaneous breathing was sufficient and the airway was patent. Patients were then transferred to the postanesthesia care unit and monitoring was continued.

**Fig. 1:**
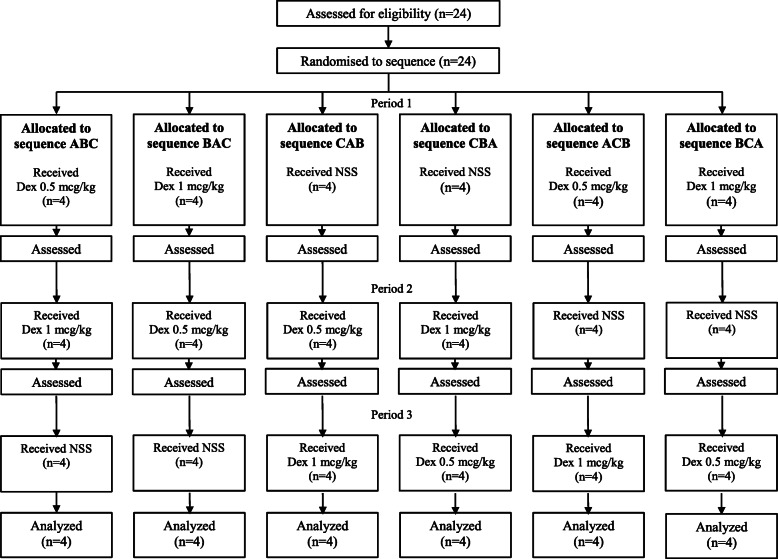
CONSORT flow diagram

Arterial blood pressure and HR were recorded immediately after ECT and then in 2.5-min intervals up to 10 min, then 5-min intervals up to 30 min. Through treatment session, acceptable blood pressure and heart rate were defined as within 20% lower or higher than each patient baseline (immediately before start premedication). If unacceptable blood pressure or heart rate persist longer than 5 min, in-charged anesthesiologist would give treatment as appropriate. The duration of EEG seizure, motor seizure and asystole were recorded. The recovery time was recorded as the time from the end of succinylcholine administration until the patient was obeying commands. The agitation degree was evaluated by a postanesthetic care nurse using a postictal agitation 4-point numeric rating scale (1 = calm or asleep, 2 = restless but calmed down when talked to, 3 = restless and required a nurse to stand next to the bed, 4 = one or more nurses were required to physically hold down the patient). The CONSORT diagram is shown in Fig. 1.

### Statistical analysis

Nominal data, such presence of underlying diseases, were summarized as number and percentage of patients. Continuous data, such as age and blood pressure, were summarized as mean ± standard deviation (SD), or median and interquartile range (IQR) based on normality of the distribution. Group comparisons were performed using a chi-square test (Fisher’s or Monte Carlo) for categorical variables and one-way ANOVA or the Kruskal–Wallis test for continuous variables. For repeated measurements and subsequent post hoc tests for systolic blood pressure, diastolic blood pressure and HR, the repeated measures ANOVA was used. The *p*-values were Bonferroni-adjusted (alpha level = 0.05/3 = 0.0167) with time as a within-group variable. SPSS 20.0 (IBM Corp., released 2011, IBM SPSS Statistics for Windows, Version 20.0. Armonk, NY) was used for statistical analysis. A p-value less than 0.05 was considered statistically significant.

## Results

**Fig. 2:**
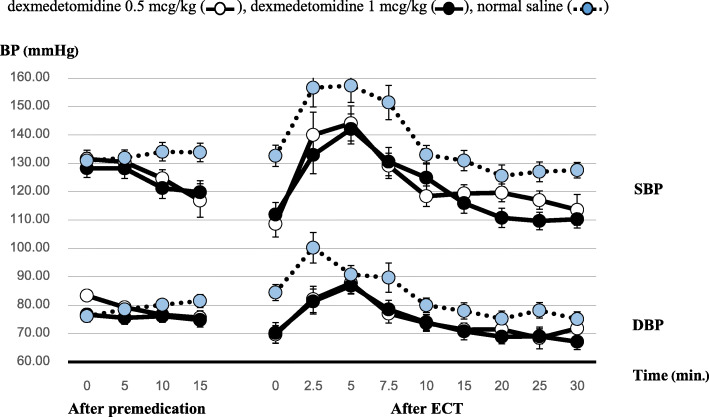
Blood pressure change in three group at different time points: dexmedetomidine 0.5 mcg/kg (), dexmedetomidine 1 mcg/kg (), normal saline () BP (mmHg)

The study included 10 male and 14 female participants. The recruitment and data collection were between July 2017 to February 2018. In total, 72 ECT sessions were evaluated from three separate treatment sessions per patient. No participants were excluded from the study due to exclusion criteria. Baseline characteristics were shown in Table 1. There were no differences in patient characteristics between the groups. Systolic and diastolic blood pressures at baseline, after drug administration, and after ECT were shown in Fig. 2.

**Table 1:** Patient demographics data

Patient characteristics	Mean ± SD
Age (y)	44.83 ± 12.71
Weight (kg)	71.71 ± 19.23
Height (cm)	162.67 ± 8.27
BMI (kg/m^2^)	27.04 ± 7.13
**Underlying**	**N (%)**
Bipolar disorder	3 (12.5%)
Schizophrenia	14 (58.33%)
Schizoaffective disorder	6 (25%)
Hypertension	5 (20.83%)
DM	4 (16.67%)
**ASA status (II/III)**	18 (75%)/6 (25%)

After administration of dexmedetomidine, systolic blood pressure of all groups was decreased from baseline within 15 min. However, the values were still within the acceptable range. Systolic blood pressure measurements in both dexmedetomidine groups were significantly lower than those in the control group at all time points after ECT (*p* = 0.002). Post hoc analysis indicated a significant difference for both the dexmedetomidine 0.5 mcg/kg group (*p* = 0.012; mean difference, − 12.27; 95% CI [− 22.41, − 2.12]) and dexmedetomidine 1 mcg/kg group (*p* = 0.003; mean difference, − 14.31; 95% CI [− 24.46, − 4.17]). The peak systolic blood pressure occurred at 5 min after ECT, and the dexmedetomidine 1 mcg/kg group had the lowest measurement at this time point (Control, 157.33 ± 28.94; Group A, 144.04 ± 30.11; Group B 142.08 ± 25.87).

Diastolic blood pressure was significantly lower in the dexmedetomidine groups compared with the control group (*p* = 0.016). Post hoc analysis indicated that diastolic blood pressure was significantly lower in only the dexmedetomidine 1 mcg/kg group compared with control group at all time points after ECT (*p* = 0.002; mean difference, − 7.55; 95% CI [− 14.19, − 0.91]).

**Fig. 3:**
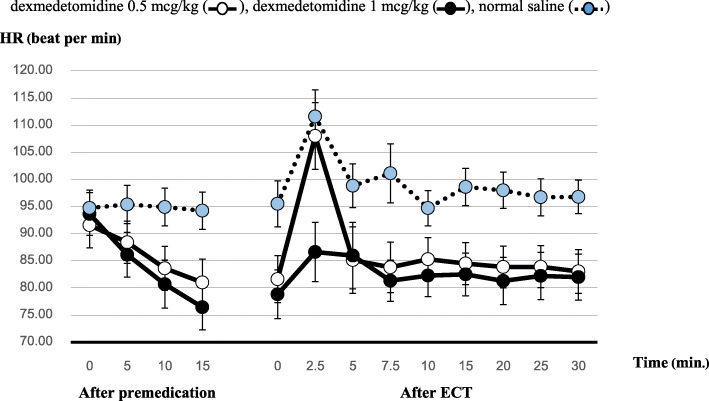
Heart rate change in three group at different time points: dexmedetomidine 0.5 mcg/kg (), dexmedetomidine 1 mcg/kg (), normal saline () HR (beat per min)

HR measurements after drug administration and ECT were shown in Fig. 3. Fifteen minutes after drug administration, HR of all groups decreased from baseline but was still higher than 70 beat/min. After ECT, HR significantly differed between the three groups (*p* = 0.012). However, when comparing between the groups, the only significant differences were between the dexmedetomidine 1 mcg/kg group and control group at all time points after ECT (*p* = 0.013; mean difference, − 14.71; 95% CI [− 26.98, − 2.44]). The carry-over effect was minimized by adequate washout period at least 24 h interval between each premedication drug before each ECT session. The carry-over covariates (period of measurement 1–3) were drop and re-run the repeated measures ANOVA for systolic, diastolic blood pressure and heart rate to check if there was significant carry-over effect. The results yielded no significant (*p* > 0.05).

Secondary outcomes were shown in Table 2. Recovery time, EEG seizure duration, asystole duration and agitation scores were similar between groups. There was a statistically significant difference in motor seizure duration between groups (*p* = 0.048), with the lowest duration measured in the dexmedetomidine 1 mcg/kg group. However, post hoc analysis indicated no significant differences with *p* > 0.0167 with Bonferroni correction; 0.05/3 pairs = 0.0167 (mean difference, − 9.208; 95% CI [− 20.9, 2.49]).

**Table 2:** Secondary outcomes compare in three groups

	DEX0.5 (n = 24)	DEX1.0 (n = 24)	NSS (*n* = 24)	P-value
ECT Energy (J)^a^	55 (40–72.5)	55 (37.5–85)	55 (35–70)	0.762
Recovery time (min)	19.17 ± 9.95	24.54 ± 13.07	18.5 ± 12.91	0.152
Motor seizure duration (min)	39.54 ± 15.38	30.92 ± 13.68	40.13 ± 13.32	0.048
EEG seizure duration (min)	53 ± 24.83	40.83 ± 16.68	52.38 ± 18.66	0.073
Asystole duration	6.79 ± 1.69	7.5 ± 2.19	8.88 ± 11.11	0.375
Agitation score ^b^				0.052
1	14 (58.33%)	19 (79.17%)	15 (62.5%)	
2	10 (41.67%)	5 (20.83%)	6 (25%)	
3	0	0	3 (12.5%)	

Data presented as mean ± standard deviation, ^a^median (interquartile range), ^b^n (%)

## Discussion

This study demonstrates that dexmedetomidine can attenuate the hemodynamic response after ECT. Either dexmedetomidine 0.5 mcg/kg or 1 mcg/kg attenuated hypertension immediately after ECT for at least 30 min, the maximum length of time measured in this study. The two dexmedetomidine doses produced similar degrees of blood pressure attenuation. However, only dexmedetomidine 1 mcg/kg decreased the tachycardia response compared to the control group, as indicated in the post hoc analysis. Dexmedetomidine administration had no statistically significant effect on recovery time, quality of ECT or motor and EEG seizure durations. Asystole duration, a complication after ECT, was also not affected by the drug administration.

Dexmedetomidine has been used in previous studies to attenuate the hemodynamic response in stress situations [13, 14]. In the ECT setting, dexmedetomidine has demonstrated various effects, which may be due to different drug doses or anesthetic regimens used in the studies [4–6, 11, 12]. In a previous study, Fu and White [4] demonstrated that a single 0.5 or 1 mcg/kg dose of dexmedetomidine before induction of anesthesia failed to decrease the peak mean arterial blood pressure (MAP) and HR after ECT. However, the varying of premedication time of dexmedetomidine administration in each patient was used in the study. Additionally, labetalol, which was used by all of the participants in their study, might influence the results. Moshiri et al. [7] used dexmedetomidine 0.5 mcg/kg, alfentanil 10 mcg/kg and saline in a cross-over study. They reported no difference in hemodynamic parameters between dexmedetomidine and saline. However, atropine was administered to all patients for bradycardia prophylaxis after using succinylcholine in their study, which might confound the hemodynamic effect of dexmedetomidine. In contrast, the cross-over study from Begec et al. [5], which used a higher dose of dexmedetomidine (1 mcg/kg), reported a significant reduction in hyperdynamic response to ECT. Another randomized controlled trial from Bagle et al. [15] reported a smaller increase in MAP and HR with dexmedetomidine 0.5 mcg/kg compared with saline. A lower dose of dexmedetomidine (0.2 mcg/kg) used in a randomized control trial by Li et al. [12] also produced a significant reduction in HR and MAP compared with saline without altering seizure duration and recovery time. In the present study, we used dexmedetomidine 0.5 mcg/kg and 1 mcg/kg, and expected the same benefit with the two different doses, but fewer side effects with the lower dose, which would also be more cost-effective. We limited the confounding factors by avoiding the use of any beta-blocker or anti-cholinergic drugs in the study protocol.

Postictal asystole after ECT has been previously reported [16–18]. The proposed mechanism is the unopposed potent vagal stimulation by mechanoreceptors after ventricular systole from depleted catecholamines [16]. Dexmedetomidine has been shown to depress sinus and atrioventricular node function [19]. It may potentiate or attenuate postictal asystole risk; therefore, postictal asystole duration is an important outcome to consider. Our study results were consistent with the cross-over study by Parikh et al. [11], which reported that dexmedetomidine 1 mcg/kg or dexmedetomidine 0.5 mcg/kg and esmolol significantly ameliorated the cardiovascular response to ECT without affecting seizure duration. From our study, the best hemodynamic effect was demonstrated in the dexmedetomidine 1 mcg/kg group. Moreover, our study showed that this cardiovascular effect can last as long as 30 min after ECT, which has not been previously reported. The peak hyperdynamic effect from ECT was observed at 2.5 to 5 min after stimulation, with the peak HR occurring earlier than the peak blood pressure. This may be explained by the non-invasive blood pressure measurements that take time to perform, whereas HR measurements were taken from real time electrocardiogram monitoring. Future studies using real time blood pressure monitoring, such as intraarterial blood pressure, should be considered to confirm the result. However, dexmedetomidine 0.5 mcg/kg failed to attenuate the peak HR response at 2.5 min after ECT compared with saline, as shown in Fig. 3.

Motor seizure duration was decreased in the dexmedetomidine 0.5 mcg/kg and 1 mcg/kg groups compared with the control group. Motor seizure duration greater than 20–25 s is usually considered as adequate treatment [20]. We found that some of the patients in the dexmedetomidine 1 mcg/kg group had motor seizure durations of less than 25 s, but none in the dexmedetomidine 0.5 mcg/kg group. This result differs from the study by Parikh et al. and may affect the quality of ECT in our patients. However, we also recorded EEG seizure duration, and EEG measures are modestly associated with clinical outcomes [21].

Asystole is an uncommon but potentially fatal complication after ECT. Post-stimulus asystole occurs just after electrical stimulation, whereas postictal asystole occurs just after seizure-induced tachycardia stops [16]. Sharp, unopposed vagal parasympathetic outflow during and immediately after an electrical stimulus results in intense but transient sinus bradycardia, with a period of sinus arrest [22]. Because of the sinus node and atrioventricular nodal depression effect that has been reported after dexmedetomidine administration [19], we were concerned that dexmedetomidine may increase the asystole duration after the ECT stimulus. However, postictal asystole was not observed in the present study, and post-stimulus asystole with duration of 6 to 9 s was observed, but did not significantly differ between the groups.

Recovery time was slightly longer in the dexmedetomidine groups compared with the saline group, but this was not statistically or clinically significant. The recovery time difference was between 5 to 10 min. Although post ECT agitation scores were similar between the groups, patients who received dexmedetomidine 1 mcg/kg seemed to be calmer and less agitated; most of the patients had an agitation score of 1 and none had agitation scores of 3–4. This favorable result is similar to many previous studies [7, 11, 23, 24].

This study had a few strengths and limitations that were worth noting. The study’s strengths were that it was a crossover, double-blind randomized controlled trial with no other medications interfering with the results of the study, such as beta-blockers or atropine. However, it is necessary to note that this study was a single-center clinical trial with a limited sample size. Multicenter or psychiatric disease-specific studies with larger sample sizes should be considered. Additionally, all of the patients in this study had previously received an ECT treatment course. We did not limit the induction agents depending on individualized effective regimens use before the study began, so patients received thiopental, propofol, or a combination. This may affect seizure duration measurements, as propofol produces shorter seizure times compared with thiopental [25].

## Conclusion

Dexmedetomidine 1 mcg/kg administered 15 min before ECT attenuated the hemodynamic response, including suppressing the systolic, diastolic and HR increases, during ECT without affecting recovery time. It also did not prolong the post-stimulus asystole duration. However, motor seizure duration was slightly shorter with this dexmedetomidine dose than with the 0.5 mcg/kg dose or saline, which may affect the quality of ECT. Decreasing the dexmedetomidine dose to 0.5 mcg/kg improved motor seizure duration compared with dexmedetomidine 1 mcg/kg, but also decreased the degree of hemodynamic attenuation.

## Data Availability

The datasets used and analyzed during the current study are available from the corresponding author upon reasonable request.

## Abbreviations

ASA: American Society of Anesthesiologist
BMI: Body mass index
DEX: Dexmedetomidine
DM: Diabetes mellitus
ECT: Electroconvulsive therapy
EEG: Electroencephalogram
HR: Heart rate
MAP: Mean arterial blood pressure
NSS: Normal saline
SBP: Systolic blood pressure
DBP: Diastolic blood pressure
